# Unsupervised spatially embedded deep representation of spatial transcriptomics

**DOI:** 10.1186/s13073-024-01283-x

**Published:** 2024-01-12

**Authors:** Hang Xu, Huazhu Fu, Yahui Long, Kok Siong Ang, Raman Sethi, Kelvin Chong, Mengwei Li, Rom Uddamvathanak, Hong Kai Lee, Jingjing Ling, Ao Chen, Ling Shao, Longqi Liu, Jinmiao Chen

**Affiliations:** 1 Singapore Immunology Network (SIgN), Agency for Science, Technology and Research (A*STAR), Singapore, 138648 Singapore; 2 Institute of High-Performance Computing (IHPC), Agency for Science, Technology and Research (A*STAR), Singapore, 138632 Singapore; 3 BGI Research-Southwest, BGI, Chongqing, 401329 China; 4 JFL-BGI STOmics Center, Jinfeng Laboratory, Chongqing, 401329 China; 5 UCAS-Terminus AI Lab, University of Chinese Academy of Sciences, Beijing, China; 6 BGI-ShenZhen, Shenzhen, 518103, China; 7 Immunology Translational Research Program, Department of Microbiology and Immunology, Yong Loo Lin School of Medicine, National University of Singapore (NUS), 5 Science Drive 2, BlkMD4, Level 3, Singapore, 117545 Singapore

**Keywords:** Spatial transcriptomics, Spatial clustering, Variational graph auto-encoder, Batch integration, Trajectory inference, Gene imputation

## Abstract

Optimal integration of transcriptomics data and associated spatial information is essential towards fully exploiting spatial transcriptomics to dissect tissue heterogeneity and map out inter-cellular communications. We present SEDR, which uses a deep autoencoder coupled with a masked self-supervised learning mechanism to construct a low-dimensional latent representation of gene expression, which is then simultaneously embedded with the corresponding spatial information through a variational graph autoencoder. SEDR achieved higher clustering performance on manually annotated 10 × Visium datasets and better scalability on high-resolution spatial transcriptomics datasets than existing methods. Additionally, we show SEDR’s ability to impute and denoise gene expression (URL: https://github.com/JinmiaoChenLab/SEDR/).

**Supplementary Information:**

The online version contains supplementary material available at 10.1186/s13073-024-01283-x.

## Background

Single-cell omics technologies enable measurements at single-cell resolution, and this has led to discoveries of new subpopulations across various tissues, in both healthy and diseased states. However, the dissociation of tissue into single cells prior to high-throughput omics data acquisition leads to cellular spatial information being lost, hindering our ability to dissect the spatial organization and intercellular interactions of individual cells. While computational tools have been developed to predict cell–cell interactions from ligand and receptor expression, they require validation using immunohistochemistry (IHC) or immunofluorescence (IF) experiments. Emerging spatial omics technologies overcome these limitations by retaining the spatial location of gene/protein expression measurements. Such spatially resolved transcriptomes of histological tissues enable the reconstruction of tissue architecture and cell–cell interactions [1–9]. This approach has proven valuable in many applications, including studies on brain disorders [2, 10], tumor microenvironments [3, 11], and embryonic development [12].

Among the currently available spatial transcriptomics approaches, in situ capturing-based technologies such as 10 × Genomics Visium and Nanostring GeoMX DSP are highly popular owing to their accessibility and ability to profile large numbers of mRNA targets within each spot. In principle, a histological section from a tissue sample is permeabilized, and the released mRNA is captured either by spatially arrayed oligos on the slide surface or by pre-hybridized RNA-target barcodes in manually defined regions of interest (ROIs). However, both technologies suffer from mRNA capture spot size limitations, with each spot covering multiple cells. To overcome this, several computational methods have been developed to deconvolve the cell mixtures of spatial spots [13–20]. Recent improvements in mRNA capture methods have led to smaller capture spots that are not greater than 10 µm in diameter. These high-resolution spatial transcriptomics methods can obtain spatially resolved transcriptomes with increased spatial fidelity without compromising the number of genes captured. They include Slide-seq [4, 7], DBiT-seq [8], Stereo-seq [5], PIXEL-seq [6], and Seq-Scope [9]. Some of these methods offer sub-cellular resolution and usually voxel binning or cell segmentation is performed to produce a gene-by-cell expression matrix for downstream analysis. Capture area sizes have also improved and increased the overall cell count throughput, necessitating new computational methods that can handle big spatial data.

Another class of methods relies on fluorescence imaging where DNA probes with attached fluorophores cyclically hybridize to cellular mRNAs, coupled with sequencing-by-ligation or sequencing-by-synthesis techniques to determine the mRNA sequence bases. Early examples of fluorescence in situ hybridization (FISH) methods had low gene coverage, but MERFISH [21] and seqFISH + [22] can achieve high gene coverage with sub-cellular resolution. Commercial vendors like 10 × Genomics, Vizgen, and Nanostring are debuting their imaging-based methods, Xenium [23], MERSCOPE, and CosMX [24], respectively, which also offer sub-cellular resolution. Similar to other in situ sequencing or barcoding-based methods with sub-cellular resolution, data preprocessing is needed to generate the desired gene-by-cell expression matrices.

When analyzing spatial transcriptomics data, combining both gene expression and spatial information to learn a discriminative representation for each cell or spot is crucial. However, established workflows such as Seurat [25] still employ pipelines designed for single-cell RNA-seq (scRNA-seq) analysis, which primarily focuses on the gene expression data and ignores the spatial arrangement of cells. Recently, several methods have been developed for spatial transcriptomics to overcome this limitation. For example, Giotto [26] and BayesSpace [27] utilize Markov random field models to detect domains with spatially coherent gene expression. stLearn [28], SpaGCN [29], DeepST [30], and STAGATE [31] adopt deep learning approaches to identify spatial domains. The latter three are based on graph neural networks, and they construct neighborhood graphs of cells or spots based on their spatial adjacency. This process can be time-consuming for datasets with large numbers of cells or spots. Some other methods combine gene expression and spatial information into a new feature matrix or latent representation that can be used for clustering and other follow-up analyses. For instance, UTAG [32] calculates an inner product between the spatial adjacency matrix and the expression matrix, returning a new matrix of spatially aggregated expression values. SpatialLDA [33] introduces a spatial regularization term to the latent Dirichlet allocation model to encourage agreement between neighboring cells and outputs spatially aware topics. SpaGene [34] detects spatially variable genes on which non-negative matrix factorization is applied to generate latent factors. Furthermore, another key challenge in spatial transcriptomics analysis is drop-out events and data sparsity, which is not yet taken into account by these existing methods.

In this work, we developed an unsupervised spatially embedded deep representation (SEDR) method for learning a low-dimensional latent representation of gene expression embedded with spatial information. The SEDR model consists of two main components, a deep masked autoencoder network for learning latent representation and a variational graph autoencoder network for embedding spatial information. These two components are optimized jointly to generate a latent representation suited for spatial transcriptomics data analysis. We applied SEDR to 10 × Genomics Visium, Slide-seq, and Stereo-seq datasets, demonstrating its ability to achieve better representations for various follow-up analysis tasks, namely clustering, visualization, trajectory inference, batch effects correction, and gene expression imputation.

## Methods

### Model structure

**Fig. 1:**
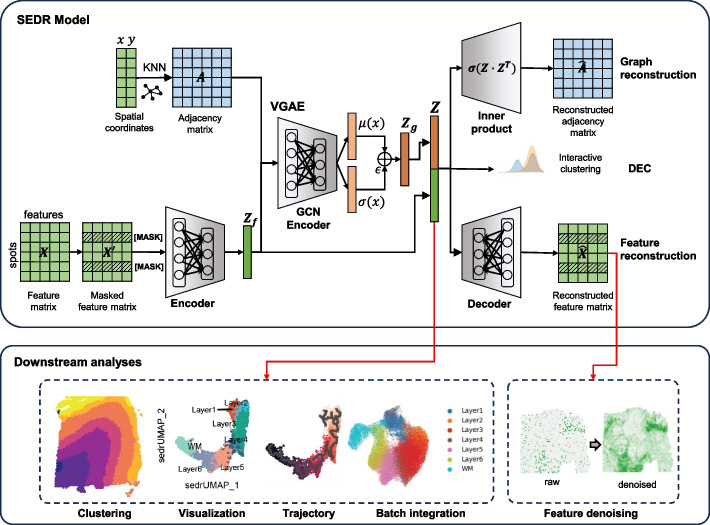
Overview of SEDR. SEDR learns a low-dimensional latent representation of gene expression embedded with spatial information by jointly training a masked self-supervised deep autoencoder and a variational graph convolutional autoencoder. The low-dimensional embedding produced by SEDR can be used for downstream visualization, spot clustering, trajectory inference, and batch effect correction. The reconstructed feature matrix can be used to impute the raw gene expression with dropouts

SEDR implements a variational graph autoencoder [35] (VGAE) coupled with a masked self-supervised learning framework to learn a latent representation from gene expression profiles and spatial information. As shown in Fig. 1, the SEDR framework contains two major components, i.e., data masking and latent representation learning. Next, we will elaborate on each component of the framework.

### Data masking

Before the latent representation learning, we first generate a masked gene expression matrix as input. The masked matrix is then fed into the deep autoencoder and the VGAE, respectively. Specifically, let $X\in {\mathbb{R}}^{n\times m}$ be a spot-by-gene expression matrix with $n$ spots and $m$ genes. We first randomly sample a subset of spots ${{\text{SP}}}_{{\text{sub}}}\subset {\text{SP}}$ and mask each of their gene expression vector ${x}_{i}\in {\mathbb{R}}^{m} (i\in {{\text{SP}}}_{{\text{sub}}})$ with a learnable vector ${\widetilde{x}}_{i}\in {\mathbb{R}}^{m}$. Thus, the input expression matrix $X$ is re-defined as the masked expression matrix ${X}{\prime}$ as follows:

$${x}_{i}{\prime}=\left\{\begin{array}{c}{\widetilde{x}}_{i}, i\in {{\text{SP}}}_{{\text{sub}}}\\ {x}_{i}, i\notin {{\text{SP}}}_{{\text{sub}}}\end{array}\right.$$

One of the main objectives of this masked self-supervised framework is to reconstruct the masked gene expressions of spots in ${{\text{SP}}}_{{\text{sub}}}$ given the remaining gene expressions ${X}{\prime}$ and spatial adjacency matrix $A$. Formally, the masked reconstruction loss is denoted as follows:

$${\mathcal{L}}_{{\text{mask}}}=\sum_{i=1}^{{{\text{SP}}}_{{\text{sub}}}}{\Vert {x}_{i}-{\widetilde{x}}_{i}\Vert }_{F}^{2}.$$

### Graph construction for spatial transcriptomics data

To create a graph representing the spot-spot spatial relationships in spatial transcriptomics data, we calculate the Euclidean distances between spots using the spatial coordinates. We then use the *K*-nearest neighbors for each spot to construct an adjacency matrix. The adjacency matrix, denoted by $A$, is a symmetric matrix, where ${A}_{ij}={A}_{ji}=1$ if $i$ and $j$ are neighbors, and 0 otherwise. To save CPU memory and running time, the adjacency matrix is stored as a sparse matrix.

### Latent representation learning

The latent representation of gene expression is learned using a deep autoencoder. The encoder part consists of two fully connected stacked layers and generates a low-dimensional representation ${Z}_{f} {\in {\mathbb{R}}}^{n\times {d}_{f}}$ from the masked gene expression matrix ${X}{\prime} \in {\mathbb{R}}^{n\times m}$. Meanwhile, the decoder part with one fully connected layer reconstructs the expression matrix $\widehat{X}\in {\mathbb{R}}^{n\times m}$ from the latent representation $Z\in {\mathbb{R}}^{n\times d}$, which is obtained by concatenating the low-dimensional representation ${Z}_{f}$ and spatial embedding ${Z}_{g} {\in {\mathbb{R}}}^{{n\times d}_{g}}$. Here, ${d}_{f},{d}_{g}, \mathrm{and }d$ are the dimensions of the low-dimensional expression representation learned by the encoder, the spatial embedding learned by the graph convolutional neural network (GCN), and the final latent representation of SEDR, respectively, with ${d=d}_{f}+{d}_{g}$. For the decoder, SEDR has two modes, clustering and gene imputation. For the clustering mode, the decoder is a GCN-based decoder which can capture more spatial information, while a plain linear decoder is used for gene imputation since it helps to avoid over-smoothing caused by a GCN-based decoder. The objective function of the deep autoencoder maximizes the similarity between the input gene and reconstructed expressions measured by the mean squared error (MSE) loss function $\sum {\left(X-\widehat{X}\right)}^{2}$.

With the adjacency matrix $A$ and its degree matrix $D$, the VGAE learns a graph embedding ${Z}_{g}$ with the following format: $g:(A,{Z}_{f})\to {Z}_{g}$, where ${Z}_{f}$ is the feature representation from the deep autoencoder. The inference part of the VGAE is parameterized by a two-layer GCN [36]:

$$g\left({Z}_{g}|{A, Z}_{f}\right)=\prod g({z}_{i}|{A, Z}_{f}), with\,g\left({z}_{i}|A,{Z}_{f}\right)=\mathcal{N}({z}_{i}|{\mu }_{i},{\text{diag}}({\sigma }_{i}^{2}))$$

where $\mu$ is the matrix of mean vectors, and $\sigma$ is the matrix of standard deviation. $\mu$ and ${\text{log}}(\sigma )$ are obtained by two-layer GCN which is defined as follows:

$${{\text{GCN}}}_{ \mu }\left({A,Z}_{f}\right)=\widetilde{A}\mathrm{ ReLU}\left(\widetilde{A}{Z}_{f}{W}_{0}\right){W}_{\mu },$$

$${{\text{GCN}}}_{{\text{log}}(\sigma )}\left({A,Z}_{f}\right)=\widetilde{A}\mathrm{ ReLU}\left(\widetilde{A}{Z}_{f}{W}_{0}\right){W}_{\sigma },$$

with weight matrices ${W}_{0}, {W}_{\mu }, {W}_{\sigma }$, and symmetrically normalized adjacency matrix $\widetilde{A}={D}^{-\frac{1}{2}}A{D}^{-\frac{1}{2}}$. In computational programming, the distribution of the spatial embedding ${Z}_{g}$ cannot be fully depicted. Instead, it is obtained by reparametrization:

$$z_g=\mu+\sigma\odot\epsilon$$

where $\epsilon \sim {\text{Normal}}(0, 1)$. After obtaining ${Z}_{g}$, merged latent representation $Z$ is obtained by concatenating ${Z}_{g}$ and ${Z}_{f}$, and the reconstructed adjacency matrix $\widehat{A}$ are generated as follows:

$$\widehat{A}=\sigma (Z\cdot {Z}^{T})$$

The objective of the VGAE is to minimize the cross-entropy (CE) loss between the input adjacency matrix $A$ and the reconstructed adjacency matrix $\widehat{A}$, while simultaneously minimizing the Kullback–Leibler (KL) divergence between $g\left({Z}_{g}|A,{Z}_{f}\right)$ and the Gaussian prior:

$$p\left({Z}_{g}\right)= {\prod }_{i}\mathcal{N}({z}_{i}|0,I)$$

### Batch effect correction for spatial transcriptomics

Spatial relationships only exist within a single spatial transcriptomic measurement; spots from different transcriptomic measurements have no direct spatial relations. Let ${A}^{k}$ and ${Z}_{f}^{k}$ denote the adjacency matrix and deep gene representation of spatial omics $k$, respectively; we then create a block-diagonal adjacency matrix ${A}^{k}$ and concatenate the deep gene representation in the spot dimension as follows:

$$A=\left[\begin{array}{ccc}{A}^{1}& \cdots & 0 \\ \vdots & \ddots & \vdots \\ 0& \cdots & {A}^{K}\end{array}\right] {Z}_{f}=\left[\begin{array}{c}{Z}_{f}^{1}\\ \vdots \\ {Z}_{f}^{K}\end{array}\right]$$

where $K$ is the number of spatial omics. Based on this formulation, the different spatial omics datasets (of potentially different sizes) are transformed into multiple graph instances in the form of one block-diagonal adjacency matrices as inputs to SEDR.

To remove batch effects and enhance the compactness of its latent representation, SEDR employs an unsupervised deep embedded clustering (DEC) method [37] to iteratively group the spots into different clusters. To initialize the cluster centers, we employ the KMeans function in scikit-learn on the learned latent representations. The number of clusters is pre-defined as a hyperparameter. With this initialization, DEC improves the clustering using an unsupervised iterative method of two steps. In the first step, a soft assignment ${q}_{ij}$ of latent point ${z}_{i}$ to cluster center ${\mu }_{j}$ is calculated using Student’s *t*-distribution:

$${q}_{ij}= \frac{{\left(1+{\left|\left|{z}_{i}-{\mu }_{j}\right|\right|}^{2}\right)}^{-1}}{{\sum }_{j\mathrm{^{\prime}}}{\left(1+{\left|\left|{z}_{i}-{\mu }_{j\mathrm{^{\prime}}}\right|\right|}^{2}\right)}^{-1}}$$

In the second step, we iteratively refine the clusters by learning from their high-confidence assignments with the help of an auxiliary target distribution $p$ based on ${q}_{ij}$:

$${p}_{ij}= \frac{{q}_{ij}^{2}/{\sum }_{i}{q}_{ij}}{{\sum }_{j\mathrm{^{\prime}}}({q}_{ij\mathrm{^{\prime}}}^{2}/{\sum }_{i}{q}_{ij\mathrm{^{\prime}}})}$$

Based on the soft assignment ${q}_{ij}$ and auxiliary target distribution ${p}_{ij}$, an objective function is defined using the KL divergence:

$$KL(P|\left|Q\right)= {\sum }_{i}{\sum }_{j}{p}_{ij}{\text{log}}\frac{{p}_{ij}}{{q}_{ij}}$$

The SEDR parameters and cluster centers are then simultaneously optimized using stochastic gradient descent (SGD) with momentum.

### Clustering

After obtaining latent representation, the results can be used to cluster for the spatial data. SEDR accepted various clustering methods. In this manuscript, mcluster (R package mclust v6.0.0) was selected because the clustering performance is high, and the number of clusters can be determined. We used Python to create a wrapped mcluster function in SEDR code.

### Data cohorts

For benchmarking SEDR with other competing methods, we utilized a human dorsolateral prefrontal cortex (DLPFC) dataset described by Kristen et al. [2]. This dataset contains 12 sections, each with 3460–4789 spots, which had been manually annotated into 7 cortical layers, namely layers 1–6 and white matter (WM). To support the ability of SEDR to denoise spatial transcriptomics data, a human ovarian cancer dataset (3493 spots), and a human lymph node dataset (4035 spots) were downloaded from the 10 × database. In addition, to demonstrate that SEDR can be applied to high-resolution spatial transcriptomics data, we used two datasets of similar sample size that were generated with Stereo-seq and Slide-seqV2, respectively [5, 7]. The Stereo-seq dataset contains 19,109 spots and 27,106 genes, and the Slide-seqV2 dataset contains 21,724 spots and 21,220 genes. In the case study of tumor heterogeneity, we downloaded a Visium dataset for human breast cancer from the 10 × database, which consists of 3798 spots and 36,601 genes.

## Results

### Overview of SEDR

SEDR learns a gene expression representation in a low-dimensional latent space with jointly embedded spatial information (Fig. 1). Given a set of spatial transcriptomics data, SEDR first learns a non-linear mapping from the gene expression space to a low-dimensional feature space using a deep autoencoder network. A masked self-supervised learning mechanism is used to enforce the encoder to capture more gene expression information through pretext tasks. Simultaneously, a variational graph autoencoder is utilized to aggregate the gene representation with the corresponding spatial neighborhood relationships to produce a spatial embedding. Next, the gene representation and spatial embedding are concatenated to form the final latent representation used to reconstruct the gene expression. Thereafter, an unsupervised deep clustering method [37] is employed to enhance the compactness of the learned latent representation. This iterative deep clustering generates a form of soft clustering that assigns cluster-specific probabilities to each spot, leveraging the inferences between cluster-specific and spot-specific representation learning. Finally, the learned latent representation can be applied towards various downstream analysis tasks, including clustering, data visualization, trajectory inference, and batch integration. Using the scaled expression as input, the reconstructed matrix can be used to impute and denoise the raw data with dropouts.

### Quantitative assessment of SEDR on human dorsolateral prefrontal cortex (DLPFC) dataset

To perform a quantitative comparison of SEDR with competing methods, we downloaded the LIBD human DLPFC data with manual annotation, acquired using the 10 × Genomics Visium spatial transcriptomics platform [2]. We chose this dataset because the human DLPFC has clear and established morphological boundaries which can serve as the ground truth [38]. We first applied the standard Seurat pipeline [25] to process and cluster spots using only gene expression profiles and set the result as the benchmarking baseline to investigate the extent to which spatial information improves spot clustering. Next, current methods that integrate gene expression with associated spatial information were used to benchmark SEDR, namely SpatialLDA [33], Giotto [26], stLearn [28], SpaGene[34], SpaGCN [29], BayesSpace [27], UTAG [32], DeepST [30], and STAGATE [31] (Additional file 1: Supplementary methods). The computational features of those methods are summarized in Table 1.

**Table 1:** Summary of features of the methods for detecting spatial domains. Compared to other methods, SEDR allows the implementation of more types of data and provides more information for downstream analyses, including latent representation and de-noised feature values. In addition, it uses GPU to accelerate calculations

Methods	Model	Resolution	Latent representation	De-noising	Batch integration	Programming	GPU
Seurat	Principal component analysis	Spot or single cell	√	×	√	R	×
SpatialLDA	Latent Dirichlet allocation	Single cell	√	×	×	Python	×
Giotto (HMRF)	Hidden Markov random field	Spot or single cell	×	×	×	R	×
stLearn	Spaital morphological gene expression normalization	Spot or single cell	√	×	√	Python	√
SpaGene	Spatial network (KNN)	Single cell	√	×	×	R	×
SpaGCN	Graph convolutional network	Spot or single cell	×	×	√	Python	×
BayesSpace	Bayesian model with a Markov random field	Spot	×	×	×	R	×
DeepST	Variational graph autoencoder	Spot or single cell	√	×	√	Python	√
STAGATE	Graph attention autoencoder	Spot or single cell	√	×	√	Python	√
UTAG	Graph + clustering	Single cell	×	×	√	Python	×
SEDR	Variational graph autoencoder + masked self-supervised	Spot or single cell	√	√	√	Python	√

**Fig. 2:**
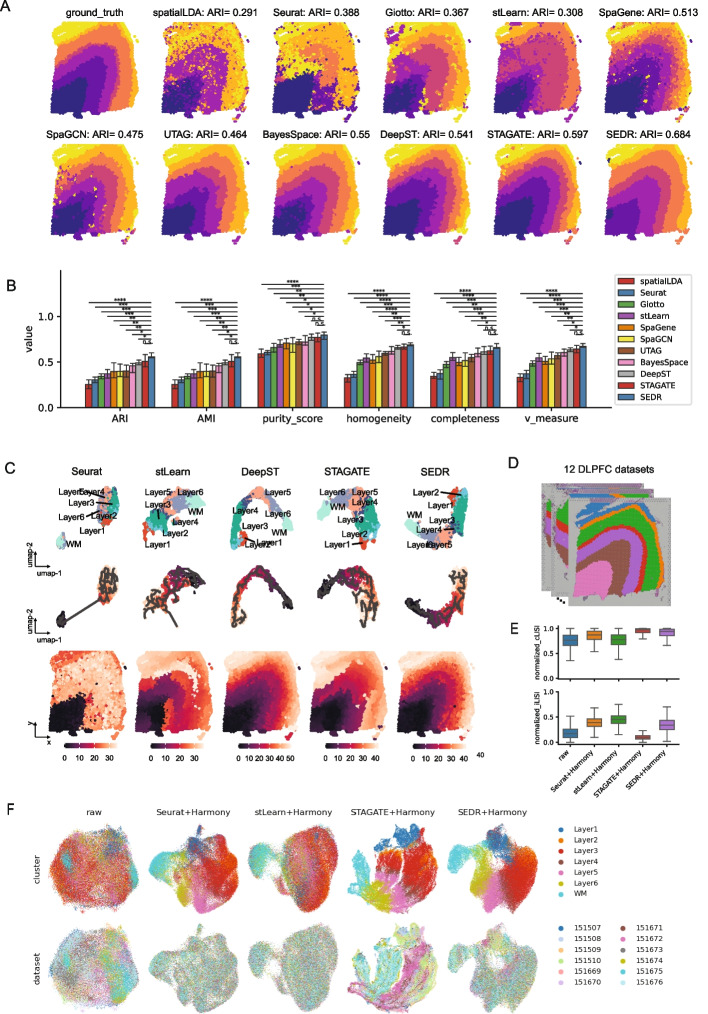
Quantitative assessment of SEDR on the human dorsolateral prefrontal cortex (DLPFC) dataset. **A** Manual annotation for the DLPFC #151673 section and clustering results of eleven methods (SpatialLDA, Seurat, Giotto, stLearn, SpaGene, SpaGCN, BayesSpace, UTAG, STAGATE, DeepST, and SEDR). **B** Barplot for the 6 performance metrics (ARI, AMI, purity score, homogeneity, completeness, v measure) on the clustering results of the DLPFC 12 sections. Notation for statistical significance testing: ^n.s.^*p*-value > 0.05, *p-value < 0.05, **p-value < 0.005, ***p-value < 0.0005, ****p-value < 0.00005. **C** UMAP and spatial visualization of Monocle 3 pseudo-time trajectories inferred with the latent representation by the tested methods of DLPFC slice #151673. UMAP plots with ground-truth labels (above), UMAP plots overlaid with Monocle 3 pseudo-time trajectories (middle), and Monocle 3 pseudo-time ordering on spatial coordinates (bottom). **D** The 12 human DLPFC sections with manual annotation. **E** Normalized cLISI and iLISI scores for DLPFC section integration results using the latent representations of four methods (Seurat, Harmony, STAGATE, SEDR). **F** UMAPs of integration results for four methods

First, we considered one cortex slice for illustration, #151,673 (Fig. 2A) with 3639 spots and 33,538 genes. We found that SEDR achieved the best performance in terms of both layer borders and adjusted rand index (ARI), followed by STAGATE (Fig. 2A). When comparing the results on all 12 DLPFC samples, we employed 6 quantitative measures, ARI, adjusted mutual information (AMI), purity score, homogeneity, completeness, and v measure. These quantitative measures reflect the goodness of matching between ground truth and predictions from different aspects (Additional file 1: Supplementary methods). For all 6 metrics, SEDR had statistically significant higher scores than competing methods, except for DeepST and STAGATE (Mann–Whitney *U* test, *p*-value < 0.05, Fig. 2B). Compared to DeepST and STAGATE, the median scores were still higher for SEDR though the difference was not statistically significant. In addition, the Silhouette score was used to measure the matching between the latent representation and the ground truth labels (Additional file 1: Supplementary methods). The Silhouette score for SEDR was the highest and only not statistically significant with respect to DeepST (Additional file 1: Fig. S1). The lack of statistical significance when compared to DeepST can be attributed to the high variance in DeepST’s Silhouette scores.

As the baseline method, the Seurat pipeline processes spatial transcriptomic data without considering the spatial information, obtaining the poorest results. Among the spatially informed methods, methods that use graph convolutional frameworks (GCN) including SpaGCN, DeepST, STAGATE, and SEDR, generally achieved better performance than others, indicating the superior ability of GCNs to integrate spatial information. When it comes to the usage of histological information, stLearn, SpaGCN, and DeepST employed the H&E image of the spots, but their performance was not outstanding, suggesting the limited utility of image information, which is potentially due to the low quality of the images. Additionally, the image processing step is inefficient in terms of running time and memory usage, especially for high-resolution images. This can make such methods unsuitable for larger spatial transcriptomics data as such data are growing in availability and scale.

To explore the robustness of SEDR and competing methods with respect to hyperparameters, we tested the top 3 methods, namely SEDR, STAGATE, and DeepST (Fig. 2) with a varying number of nearest neighbors (*K*), which determines the level of local spatial smoothing. There was a slight fluctuation of ARI scores for all 3 methods on the 12 DLPFC sections with different *K* values (Additional file 1: Fig. S2). But for each *K*, SEDR achieved the best ARI score over the other methods. Furthermore, even the lowest median ARI score (0.532 when *K* = 14) of SEDR was higher than the best score of STAGATE (0.50 when *k* = 18).

We next tested SEDR’s low-dimensional representation features in trajectory inference [39]. Monocle3 [40] was employed to perform trajectory inference on the same DLPFC slice (#151,673) with the low-dimensional representations from Seurat, stLearn, DeepST, STAGATE, and SEDR (Additional file 1: Supplementary methods). In the UMAP plot, the RNA-only representation (Seurat) clearly segregated the WM as a separate cluster from the other layers and the cortical layers showed mixing with no clear ordering according to their developmental trajectory (Fig. 2C, top). In contrast, the spatially informed methods’ latent embeddings mostly recapitulated the ordering from the WM to layer 6 and sequentially to layer 1. Setting WM as the root, the pseudo-time of trajectory was computed with Monocle. With DeepST and SEDR’s latent representations, Monocle could accurately infer the “inside out” developmental trajectory. By plotting trajectories on the original spatial positions, we observed a strong correlation between the ordering of pseudotime and the physical location of cortical layers (Fig. 2C, middle and bottom). Comparatively, the trajectories from stLearn and STAGATE’s embeddings, while improving over the RNA-only inference, still showed incorrect layer locations based on the inferred orderings. This demonstrated that incorporating spatial information into pseudo-time trajectory inference can be beneficial and SEDR’s latent representation is well-suited for this application [38, 41, 42].

### SEDR corrects for batch effects

The proliferation of spatial omics applications is generating ever-increasing volumes of spatially resolved omics data across different labs. However, differences in protocols and technologies complicate comparisons and data integration when trying to achieve consensus on spatially resolved tissue atlases. As with scRNA-Seq, removing batch effects in spatial omics datasets is a significant challenge. The deep embedded clustering (DEC) loss function employed in the SEDR model helps retain biological variations while reducing technical variations. Here, we tested the use of joint embeddings across multiple batches and projected them into a shared latent space.

We evaluated three methods, SEDR, stLearn, and STAGATE, using their latent representations of the DLPFC dataset in batch integration (Fig. 2D, E). To establish a baseline, we used Seurat to calculate the PCA embeddings for raw data (Fig. 2F). For batch integration, we employed Harmony due to its superior performance in scRNA-seq data integration [43]. With Seurat’s PCA embeddings, Harmony mixed the batches evenly but also mixed the cortical layers together (e.g., layers 1 and 2, Seurat UMAP, Fig. 2F). With stLearn’s embeddings, the cortical layers were also well-mixed. For STAGATE and SEDR’s embeddings, the different cortical layers’ cells showed clear separation with the developmental ordering visible. However, STAGATE’s integrated output showed the batches to be integrated into two major clusters. In contrast, the batch mixing was much more even in SEDR’s output. We also assessed the batch integration results with the integration LISI (iLISI) and cell type LISI (cLISI) metrics for all 12 slices (Fig. 2E, Additional file 1: Supplementary methods). In terms of cLISI, stLearn was the poorest with a score similar to the uncorrected data, and this corroborated the visual inspection of the UMAP. In terms of iLISI, the SEDR-derived integration was third (Mann–Whitney *U* test < 0.05), again matching the observations made on the plotted UMAPs. Taking both cell type separation and batch integration into consideration, we consider SEDR to be the overall best-performing method.

The integration results also suggested that the inclusion of spatial information within embeddings can have variable results. In the case of SEDR’s embeddings, the spatial information was integrated such that it improved the cortical layer cell label separation over the typical PCA embedding. On the other hand, it may have interfered with the batch integration as in STAGATE’s case. Overall, this example demonstrated that the combination of SEDR and Harmony can be effective for batch integration of spatial transcriptomics data.

### Effective noise removal and imputation of spatial transcriptomics with SEDR

Spatial transcriptomics offers unprecedented opportunities in dissecting tissue heterogeneity but suffers from measurement noise including dropouts. Consequently, effective imputation with noise removal can help reveal spatially resolved features within the data. With masked self-supervised learning, SEDR can construct a denoised and imputed gene expression matrix, which cannot be achieved by other competing methods for learning latent representations. Instead of using principal components (PCs) as input for clustering tasks, SEDR uses the processed gene expression matrix as input and generates the reconstructed expression matrix with the decoder module.

**Fig. 3:**
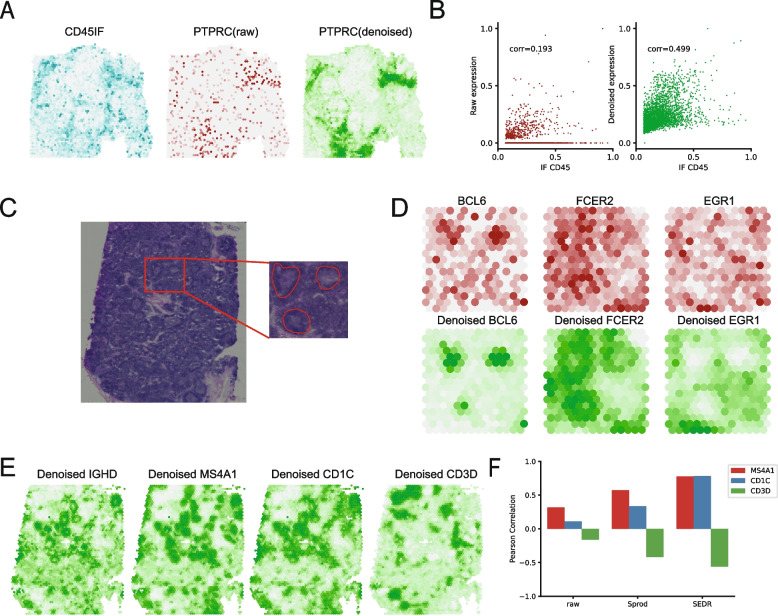
Denoised gene expression with SEDR. **A** Immunofluorescence (IF) values, raw RNA-seq counts, and denoised gene expression by SEDR for PTPRC (CD45) on the ovarian cancer dataset. **B** Scatter plot of scaled IF values with raw counts and de-noised PTPRC expression by SEDR. **C** H&E image with identified germinal centers (GCs) in the lymph node tissue sample. **D** Denoised expression of three marker genes within the selected GCs. BCL6, FCER2, and EGR1 are the markers for different regions of GCs. **E** Denoised expression of IGHD and three genes that are correlated with IGHD: MS4A1 and CD1C are positively correlated with IGHD while CD3D is negatively correlated with IGHD. **F** Pearson correlation between IGHD and the correlated genes (MS4A1, CD1C, CD3D) as measured by raw counts, de-noised value by Sprod, and de-noised value by SEDR

On the 10 × Genomics Visium human ovarian cancer dataset (downloaded from the 10 × website) [44] (Fig. 3A, B), the PTPRC (CD45) gene expression showed poor correlation (0.193) with the protein expression from immunofluorescent (IF) staining. This is primarily explained by the high levels of dropouts for PTPRC expression in the data. Using SEDR, we successfully imputed the PTPRC expression to attain a higher correlation of 0.499. Visually, we can observe the imputed region in the top left which was captured by IF but not found in the original data, indicating that such denoising is not simply spatial smoothing.

To further investigate SEDR’s denoising and imputation capabilities, we next applied SEDR to a healthy human lymph node dataset (Fig. 3C) [44]. The lymph node contains substructures called germinal centers (GCs), which can be identified through H&E staining. We first outlined three GCs and plotted the denoised values of three gene markers for substructures within the GC. As shown in Fig. 3D, the denoised BCL6, FCER2, and EGR1 expressions better delineated the germinal center structures compared to the raw expressions. The gene BCL6 marks the mature B cells found in the center of the GC, while FCER2 is a marker for naïve B cells located at the marginal zone of the follicle containing the GC, and EGR1 marks activated B cells found outside the follicles [45]. We also assessed the gene–gene correlations before and after the denoising. IGHD is known to positively correlate with MS4A1 and CD1C and negatively with CD3D. We found SEDR’s denoised values to show stronger expected gene–gene correlations than the raw and the Sprod [44] denoised values (Fig. 3E, F).

### SEDR can handle high-resolution spatial transcriptomics

**Fig. 4:**
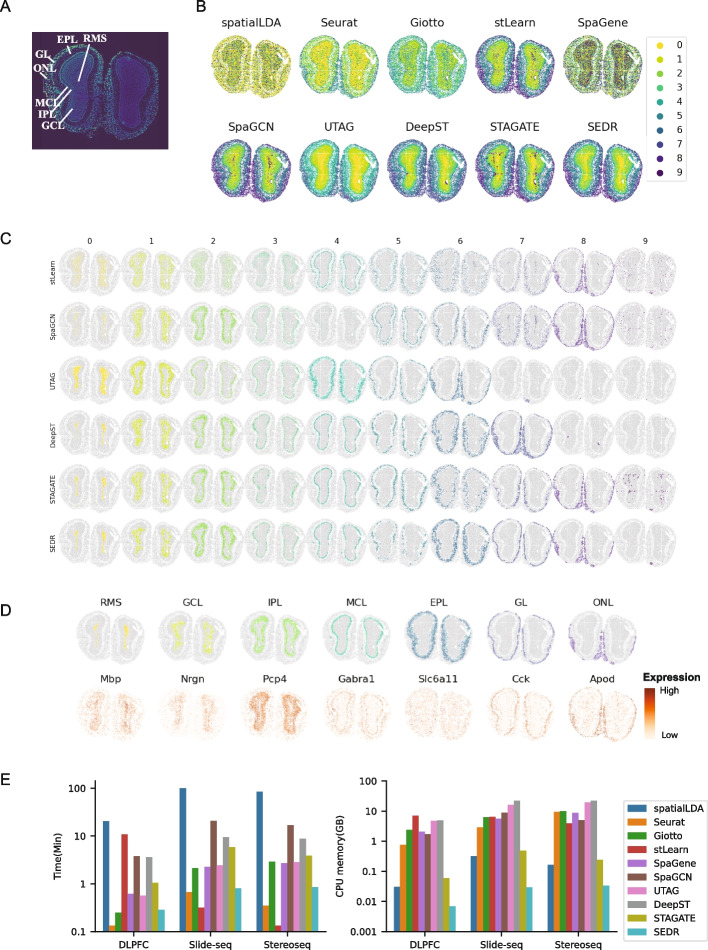
Application of SEDR on Stereo-seq dataset. **A** Laminar organization of a DAPI-stained mouse olfactory bulb. **B** Unsupervised clustering results from SEDR and nine competing methods on the olfactory bulb Stereo-seq data. **C** Visualization of individual identified clusters for selected methods (stLearn, SpaGCN, UTAG, DeepST, STAGATE, and SEDR). **D** Predicted olfactory bulb layers and corresponding marker genes. **E** Computational efficiency of ten methods tested on slice #151673 of the DLPFC set, and Stereo-seq data and Slide-seq data of mouse olfactory bulb. Left: run time of methods. Right: CPU memory usage of methods

Newly emerging methods such as Stereo-seq [5], PIXEL-Seq [6], and Seq-Scope [9] can achieve sub-micrometer and thus subcellular resolution. With continued technology advancement, the spatial resolution and number of cells detected per tissue will significantly improve, producing large datasets with high throughput. As such, we evaluated SEDR’s performance on Stereo-seq and Slide-seq data of mouse olfactory bulb tissues. We first consider the Stereo-seq data with 19,109 spots and 27,106 genes. The coronal section of a mouse olfactory bulb can be divided into 7 layers, the olfactory nerve layer (ONL), glomerular layer (GL), external plexiform layer (EPL), mitral cell layer (MCL), internal plexiform layer (IPL), granule cell layer (GCL), and rostral migratory stream (RMS) (Fig. 4A). We performed dimension reduction and unsupervised clustering using 10 methods to computationally reconstruct the spatial distribution of tissues within the olfactory bulb. With this data, stLearn, SpaGCN, UTAG, DeepST, STAGATE, and SEDR all produced distinct clusters that match the layers in the olfactory bulb, consistent with the annotated staining. (Fig. 4A, B). We further plotted the individual clusters per method to visualize their differences (Fig. 4C). For stLearn, SpaGCN, and STAGATE, they were unable to fully remove the technical noise from Stereo-seq seen near the core of the olfactory bulb, as visible in their cluster 9. Overall, we found DeepST, STAGATE, and SEDR to be competitive with the most similar clusters which also well matched the markers of the different tissue domains within the olfactory bulb (Fig. 4D). Namely, cluster 0 corresponded to the RMS, cluster 1 to GCL, cluster 2 to IPL, clusters 3 and 4 to MCL, clusters 5 and 6 to EPL, cluster 7 to GL, and cluster 8 to ONL. We also applied the methods to the Slide-seq data acquired from the mouse olfactory bulb with 21,724 spots and 21,220 genes. The clusters obtained are provided in Additional file 1: Fig. S3. Similar to the Stereo-seq data, stLearn, SpaGCN, UTAG, DeepST, STAGATE, and SEDR showed good performance.

With the increasing sample size of high-resolution spatial transcriptomics data, the scalability of processing methods becomes correspondingly more and more important. Here, we tested the run time and memory usage of the ten methods. As shown in Fig. 4E, SEDR required less time than other methods except for Seurat and stLearn. It is reasonable for Seurat to require less run time as the Seurat pipeline only performed the baseline PCA dimension reduction of the gene expression matrix and ignored spatial information. For stLearn, it required much more time on the 10 × Visium DLPFC but less time on Slide-seq and Stereo-seq data, because its pipeline for 10 × Genomics Visium data contains a time-consuming image processing step, while the pipeline for the other two datasets does not contain such step. For CPU memory usage, SEDR required less CPU memory than all other methods, which can be explained by the use of a sparse matrix in its graph construction and computational steps involved in graph multiplication, greatly reducing the CPU memory requirement. We also compared SEDR with DeepST and STAGATE for GPU memory usage. SEDR also required less GPU memory than the other two methods (Additional file 1: Fig. S4A). To further demonstrate SEDR’s capacity in handling large datasets, we created a series of augmented datasets based on the mouse olfactory bulb Slide-seq data, which contained 1/2, 1, 2, and 4 times the number of spots compared to the original data (20,000 spots). The results demonstrated that the cost of time, CPU, and GPU memory were still acceptable even in the case of 4 × data size (80,000 spots) (Additional file 1: Fig. S4B).

### Dissecting tumor heterogeneity and immune microenvironments using SEDR

**Fig. 5:**
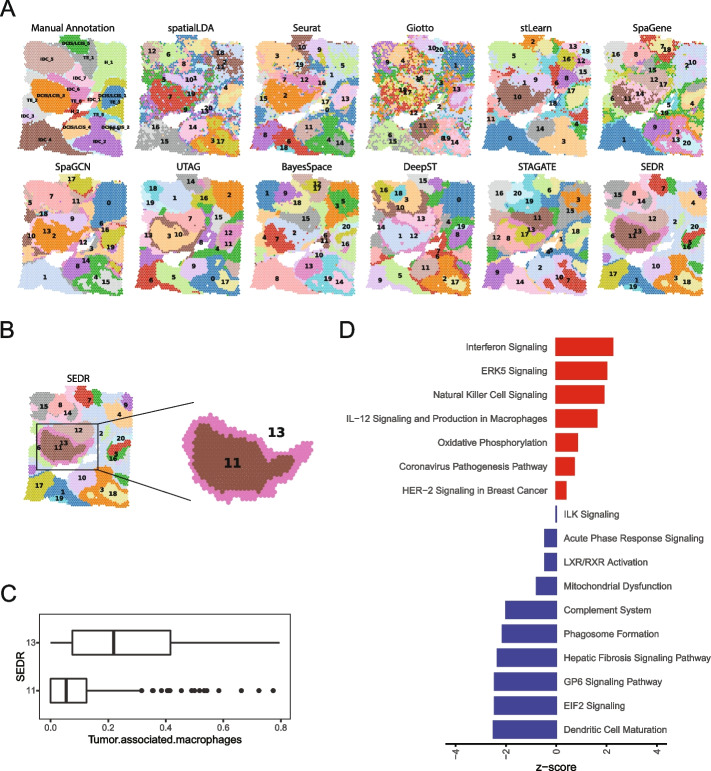
Application of SEDR on 10 × Visium spatial transcriptomics data of human breast cancer. **A** Manual pathology labeling based on H&E staining (annotation) and clustering results of eleven methods. **B** SEDR clusters 11 (core) and 13 (out ring) that captured the annotated DCIS/LCIS_3 region. **C** Percentage of tumor-associated macrophages (TAMs) in clusters 11 and 13. **D** Enriched pathways for differentially expressed genes identified between clusters 11 and 13

Intratumoral heterogeneity in cancer complicates effective treatment formulations and is associated with poor survival prospects [46]. Spatial transcriptomics offers advantages over scRNA-seq at dissecting and characterizing intratumoral heterogeneity and tumor-immune crosstalk by retaining the spatial information that can help reconstruct spatially distributed domains and distance-dependent interactions. In this example, we tested SEDR on the 10 × Genomics Visium spatial transcriptomics acquired data for human breast cancer, which is known for its high intratumoral and intertumoral differences [47]. To aid in interpreting the SEDR’s results, we performed manual pathology labeling based on the H&E staining. It should be noted that, unlike the cerebral cortex which has clear and established morphological boundaries, tumor tissues are highly heterogeneous and encompass complex microenvironments; manual labeling solely based on tumor morphology is inadequate for characterizing such complexity. Based on the pathological features, we first manually segmented the histology image into 20 regions, which we then grouped into 4 main morphotypes: ductal carcinoma in situ/lobular carcinoma in situ (DCIS/LCIS), healthy tissue (Healthy), invasive ductal carcinoma (IDC), and tumor surrounding regions with low features of malignancy (Tumor edge) (Fig. 5A, Additional file 1: Fig. S5A).

To detect spatial domains, eleven methods, namely SpatialLDA, Seurat, Giotto, stLearn, SpaGene, SpaGCN, UTAG, BayesSpace, DeepST, STAGATE, and SEDR, were used to produce the same number of clusters (20). Visually, all methods agreed with the manual annotations at the macroscopic level (Fig. 5A). Giotto’s clusters showed the highest levels of fragmentation, more than Seurat’s, a non-spatially aware method. Notably, SEDR, UTAG, and DeepST divided the tumor region DCIS/LCIS_3 into an outer “ring” and a tumor core, while BayesSpace, SpaGene, and STAGATE divided the region into two halves.

To explore the cell types in SEDR cluster 11 (tumor core) and cluster 13 (tumor edge) in SEDR clustering results (Fig. 5B), Seurat 3 was employed to deconvolute spatial data against the scRNA-seq reference data for human breast [48] (Additional file 1: Supplementary methods). For each spot, we obtained a composition vector for cell types (Additional file 1: Fig. S5B). Interestingly, we found that cluster 13 contains significantly more tumor-associated macrophages (TAMs) than cluster 11 (Fig. 5C). TAM infiltration is known to be strongly associated with poor survival rate in solid tumor patients due to its promotion of tumor angiogenesis and induction of tumor migration, invasion, and metastasis [49, 50].

We also performed differential expression analysis followed by pathway enrichment analysis (Fig. 5D, Additional file 1: Supplementary methods). In cluster 11, we observed the upregulation of interferon signaling pathways (IFIT1, IFITM1, IFITM3, and TAP1) and NK or neutrophil activities (FCGR3B and TNFSF10) (Fig. 5D, Additional file 1: Fig. S6). In addition, RHOB was upregulated in this region, pointing towards reduced metastatic potential [51]. Therefore, cluster 11 represented a region where cancer growth was limited by pro-inflammatory immune responses. On the other hand, in cluster 13, we observed the presence of TAMs (Additional file 1: Fig. S5B), memory B cells (IGHG1, IGHG3, IGHG4, IGLC2, and IGLC3), and fibroblasts (COL1A1, COL1A2, COL3A1, COL5A1, COL6A1, COL6A2, and FN1) (Additional file 1: Fig. S6). Upregulated cathepsin activity (CTSB, CTSD, and CTSZ) and complement pathway (C1QA, C1S) indicated pro-tumor activity by the TAMs in this region [52–54]. Overall, cluster 13 represented a region with an immune-suppressed pro-tumor microenvironment and a high potential for cancer metastasis.

In summary, SEDR analysis dissected intratumoral heterogeneity within visually homogeneous tumor regions and revealed the tumor outer ring (cluster 13) with TAM infiltration and cancer-associated fibroblasts (CAFs), both of which have been reported to facilitate tumor spread [55, 56].

## Discussion

Cell type heterogeneity is a feature of both healthy and diseased tissue. Capturing this heterogeneity, coupled with its spatial arrangement in the tissue, is crucial when studying the roles of cells and their crosstalk. Spatial omics technologies represent the state-of-the-art approaches for capturing omics data with corresponding spatial information from tissue samples. In this paper, we introduced SEDR, which leverages cutting-edge graph neural network techniques to achieve a better representation of spatial omics data that can be used for clustering and further downstream analyses. SEDR first learns a low-dimensional latent space representation of the transcriptome information with a deep autoencoder network coupled with masked self-supervised learning, which is then aggregated with spatial neighborhood information by a variational graph autoencoder to create a spatial embedding. This spatial embedding is then concatenated with the encoded gene expression to reconstruct the final gene expression for further analyses. We first demonstrated SEDR’s efficacy in delineating the different cerebral cortex layers with higher clarity than competing methods and recapitulating the associated development order by using the joint latent representation with Monocle 3. We also demonstrated SEDR’s ability in data imputation with human ovarian cancer and health lymph node tissue data acquired with the 10 × Genomics Visium technology. SEDR is not restricted to Visium-acquired data, as we also showed its efficacy on high-resolution Stereo-seq and Slide-seq data, being able to more accurately recover tissue domains compared to competing methods.

To enhance the analytical power and resolution of spatial omics, we need to integrate multiple datasets from the same tissue. Similar to single-cell transcriptomic data, spatial omics datasets generated in different batches also contain batch-specific systematic variations that present a challenge to batch-effect removal and data integration. In our study, we demonstrated that by combining SEDR and Harmony, we were able to effectively remove batch effects present.

Spatial omics technologies such as Stereo-seq are able to measure a large number of spots in a single experiment through high spatial resolutions and large tissue sizes. Based on current trends, we expect to see ever-increasing throughput from spatial omics experiments, which will result in spatial omics big data that poses significant challenges to data analysis and integration. Computational methods that employ GCNs require the entire graph to be loaded into GPU memory, which inhibits their application to very large datasets. Currently, the current version of SEDR is the most CPU and GPU memory efficient and second fastest among methods that do not employ image processing. To enable SEDR to scale with larger datasets, we will further improve the memory efficiency of SEDR using a GCN mini-batch or parallel techniques to construct large-scale graphs for spatial omics data of high throughput and resolution. Furthermore, technologies with a capture spot size smaller than the diameter of a cell will also require new computational methods that can accurately delineate cells based on capture spots. We plan to integrate cell segmentation based on H&E or DAPI staining images into the SEDR workflow to handle such data.

The current SEDR methodology employs gene expression and spatial information and does not make use of histology images. Contemporary methods such as SpaGCN and stLearn use histological images as input, but in a suboptimal fashion, as demonstrated in our study. Specifically, SpaGCN utilizes histology image pixels as features by calculating the mean color values from the RGB channels directly. However, the pixel values are easily affected by noise and cannot provide semantic features for cell analysis. A potentially more effective approach is to adopt a deep CNN model which can learn high-level representations of histology images. stLearn introduces a deep learning model to extract the image features of spots and integrates them with the spatial location and gene expression. However, stLearn employs a model pre-trained on natural images which is not fine-tuned for histology images. In the future, we will incorporate histology images as an additional modality into the SEDR model. We will employ an image autoencoder network to first learn image features, followed by joint learning of the latent representation by integrating gene expression, image morphology, and spatial information.

In summary, SEDR is a promising new approach that builds an integrated representation of spots using both transcriptomic data and spatial coordinates. SEDR-derived low-dimensional embedding enables more accurate clustering, trajectory inference, batch effect correction, gene expression imputation, and denoising. Our model is also able to handle spatial transcriptomics with capture spot sizes ranging from 50 µm to less than 1 µm. Furthermore, we applied SEDR on a human breast cancer sample to reveal heterogeneous sub-regions within the seemingly homogeneous tumor region and shed light on the role of immune microenvironments on tumor invasiveness.

## Conclusions

In this work, we introduce SEDR as a novel method that processes spatial transcriptomics data to derive deep representation, which benefits various downstream analyses, including spatial clustering, batch integration, trajectory inference, gene expression imputation, and denoising. To obtain more accurate and informative low-dimensional representations from spatial transcriptomics data, SEDR integrates spatial information with RNA-seq data using a variational graph autoencoder, which improves the embedding results significantly compared to the one purely based on RNA-seq data.

We conclude by discussing the future development of SEDR. Although SEDR works well on data generated by 10 × Genomics Visium, Slide-seq, and Stereo-seq, the ability for it to integrate other types of spatial transcriptomics data is not fully investigated, especially for the technologies based on multiplexed imaging, such as SeqFISH [22] and MERFISH [57], which are different from spot-based ones in terms of data format, resolution, and data quality. We plan to upgrade SEDR to enable it to analyze more data types. We also plan to employ a mini-batch strategy to train models on part of the graph instead of the whole dataset to further enhance its scalability. Furthermore, we will also upgrade SEDR to include more downstream analyses and visualization methods to make it more user-friendly. 

### Supplementary Information

**Additional file 1: Supplementary methods. Fig. S1.** Silhouette score for 5 methods on 12 DLPFC sections. n.s.: *p*-value > 0.05, *: *p*-value < 0.05, **:* p*-value < 0.005, ***:* p*-value < 0.0005, ****:* p*-value < 0.00005. **Fig. S2.** ARI boxplot for 3 methods on 12 DLPFC datasets with different K (number of nearest neighbors). **Fig. S3.** Unsupervised clustering results for SEDR and competing methods on olfactory bulb Slide-seq data. **Fig. S4.** Computational requirements of SEDR. A) GPU memory usage for DeepST, STAGATE and SEDR when processing DLPFC, mouse olfactory bulb Slide-seq and Stereo-seq data. B) Time, CPU memory and GPU memory costed by SEDR on simulated data. Simulated data is generated with Slide-seq data (20000 spots) by randomly selecting ½, 1, 2, 4 times of the spots as the raw data. **Fig. S5.** Human breast cancer histology and cell type mixtures of spatial spots. A) H&E staining. B) Probability of cell types for spots that was predicted by Seurat. **Fig S6.** Differentially expressed genes (DEGs) between SEDR cluster 11 and cluster 13 in human breast cancer data.

## Data Availability

(1) The LIBD human dorsolateral prefrontal cortex (DLPFC) dataset was downloaded from https://research.libd.org/globus/jhpce_HumanPilot10x/index.html [2]. (2) The 10 × Genomics Visium spatial transcriptomics and Stereo-seq of mouse olfactory bulb datasets are downloadable from https://github.com/JinmiaoChenLab/SEDR_analyses/tree/master/data [58]. (3) The Slide-seqV2 data can be downloaded from https://singlecell.broadinstitute.org/single_cell/study/SCP815/ [7]. (4) Scripts to reproduce the benchmarking in Fig. 2 are available at https://github.com/JinmiaoChenLab/SEDR_analyses/ [59]. (5) SEDR was written in Python using the PyTorch library. An open-source implementation of SEDR has been released at https://github.com/JinmiaoChenLab/SEDR/ [60].
