# Supplementary material for: Unsupervised spatially embedded deep representation of spatial transcriptomics
Source: Genome Med. 2024 Jan 12;16:12. doi: 10.1186/s13073-024-01283-x (PMC10790257; doi:10.1186/s13073-024-01283-x)
Supplement: Supplementary file 1 — Additional file 1: Supplementary methods. Fig. S1. Silhouette score for 5 methods on 12 DLPFC sections. n.s.: p-value > 0.05, *: p-value < 0.05, **: p-value < 0.005, ***: p-value < 0.0005, ****: p-value < 0.00005. Fig. S2. ARI boxplot for 3 methods on 12 DLPFC datasets with different K (number of nearest neighbors). Fig. S3. Unsupervised clustering results for SEDR and competing methods on olfactory bulb Slide-seq data. Fig. S4. Computational requirements of SEDR. A) GPU memory usage for DeepST, STAGATE and SEDR when processing DLPFC, mouse olfactory bulb Slide-seq and Stereo-seq data. B) Time, CPU memory and GPU memory costed by SEDR on simulated data. Simulated data is generated with Slide-seq data (20000 spots) by randomly selecting ½, 1, 2, 4 times of the spots as the raw data. Fig. S5. Human breast cancer histology and cell type mixtures of spatial spots. A) H&E staining. B) Probability of cell types for spots that was predicted by Seurat. Fig S6. Differentially expressed genes (DEGs) between SEDR cluster 11 and cluster 13 in human breast cancer data. [file 13073_2024_1283_MOESM1_ESM.docx]

**Supplementary materials**

**Supplementary methods**

Data preprocessing

Baseline and competing methods

Evaluation metrics for clustering

Several downstream analyses

Methods for breast cancer data analyses

**Supplementary figures**

Fig S1. Silhouette score for 5 methods on 12 DLPFC sections.

Fig S2. ARI boxplot for 3 methods on 12 DLPFC datasets with different K (number of nearest neighbors).

Fig S3. Unsupervised clustering results for SEDR and competing methods on olfactory bulb Slide-seq data.

Fig S4. Computational requirements of SEDR.

Fig S5. Human breast cancer histology and cell type mixtures of spatial spots.

Fig S6. Differentially expressed genes (DEGs) between SEDR cluster 11 and cluster 13 in human breast cancer data.

**Supplementary methods**

**Data Preprocessing**

***Common processing pipeline using Scanpy***

SEDR takes spatial transcriptomic gene expressions and spatial coordinates as inputs. The raw gene expression counts are first normalized by their respective library sizes (using the normalize_total function in Scanpy (v.1.9.3)), with very highly expressed genes excluded when computing the normalization factor (size factor) for each spot [35]. Thereafter, all gene expression profiles are scaled to zero mean and unit variance. PCA is then applied to extract the first 200 principal components to generate the initial gene expression matrix. For most downstream applications, PCs are the recommended input for SEDR. SEDR also accepts scaled gene expression as input when imputing gene expression data.

***Stereo-seq data***

Fastq files were generated using the MGI DNBSEQ-Tx sequencer. Coordinate identities (CIDs) and unique molecular identifiers (UMIs) were encoded in the forward reads (CID: 1-25bp, UMI: 26-35bp), while the reverse reads consisted of the cDNA sequences. CID sequences in the forward reads were first mapped to the designed coordinates of the *in-situ* capture chip, allowing one base mismatch to account for sequencing and PCR errors. Reads with UMIs containing either N bases or more than two bases with quality scores lower than 10 were filtered out. The CIDs and UMIs associated with each read were appended to each read header. Retained reads were then aligned to the reference genome (mm10) using STAR [1], and mapped reads with MAPQ ≥10 were counted and annotated using an in-house script (available at https://github.com/BGIResearch/handleBam). UMIs with the same CIDs and gene loci collapsed together, allowing for one mismatch for sequencing and PCR errors, to give the final gene expression matrix.

**Baseline and competing methods**

To evaluate the clustering performance of SEDR, 11 state-of-the-art methods were used to benchmark SEDR on different datasets. Seurat [2] was used as baseline method because it only uses gene expression data. Other methods all utilized spatial information, namely SpatialLDA [3], Giotto [4], stLearn [5], SpaGene [6], SpaGCN [7], UTAG [8], BayesSpace [9], DeepST [10], and STAGATE [11]. To evaluate the efficacy of SEDR on high-resolution spatial transcriptomics data such as Stereo-seq and Slide-seq, all methods except BayesSpace were tested as we failed to run it on these data types. A more detailed comparison of the methods’ features can be found in Table 1. Here we briefly describe the usage of each method.

***Seurat*.** Seurat was designed to analyze single cell data, which is quite different from spatial transcriptomics data, either 10x Genomics Visium data or high-resolution spatial data. Here it was used as a baseline method to show the advantage of incorporating spatial information. Therefore, we only ran the basic processing steps of it, including quality control (QC), gene normalization, and principal component analysis (PCA). The top 100 principal components (PCs) were used to calculate the shared nearest neighbors (SNNs), which were then used to identify clusters with the Louvain clustering algorithm. Because the Louvain algorithm cannot directly produce a specified number of clusters, different clustering resolutions were tested to obtain the same number of clusters as the ground truth.

***SpatialLDA.*** SpatialLDA uses the documents-words-topics paradigm to apply latent Dirichlet allocation to multiplexed image data. We set n_topics to 30 and retained the other parameters at their default settings when generating the topics for each dataset. The topics were then clustered using the mclust package in R.

***Giotto*.** Gitto is a framework to process and analyze spatial transcriptomics data. It implements a hidden Markov Field (HMRF) algorithm to perform spatial clustering. We followed the tutorial of Giotto and used the default parameters for all spatial datasets. For data with ground truth (such as the DLPFC dataset), the parameter *k* was set to the number of clusters in the ground truth.

***stLearn*.** stLearn uses the histology image and spot position to help the construction of the Spatial Morphological gene Expression (SME) weighting matrix, which is used to do SME-based gene normalization. To run stLearn, we referred to the tutorial and used its default parameters to run it on both spot-based and single cell spatial transcriptomics data. We noticed that in their tutorial, for high resolution spatial data, such as Slide-seq, it does not include the use of histology in their tutorial. We also followed this setting in our manuscript.

***SpaGene***. SpaGene is a model free algorithm to detect spatially variable genes on which non-negative matrix factorization is applied. We used the default parameters to generate 30 patterns for each dataset. The patterns were then clustered with the mclust algorithm.

***SpaGCN***. SpaGCN combines spatial locations, histological similarities, and gene expression to construct a graph and uses a graph convolutional network (GCN) to cluster the spots. As suggested by the tutorials, a 40x40 pixel snapshot of the histology image was cropped from the H&E image. Adjacency matrix was then calculated with histology set to True. The GCN model was then trained for 200 epochs with the learning rate set to 0.05.

***BayesSpace*.** BayesSpace implements a Bayesian approach to start from a HMRF-derived prior and update the clustering results until it converges. To run BayesSpace, the number of PCs was set as 15, the smoothing parameter as 3. The model was trained for 50000 iterations.

***UTAG***. UTAG is designed for spatial transcriptomics data generated with multiplexed imaging, which has become popular in recent years. It implements a graph approach to combine spatial information with gene expression data, followed by clustering. We ran UTAG with max distance as 60 and used the Leiden algorithm to do clustering.

***DeepST***. DeepST utilizes morphological information, spatial distance, and gene expression to generate an enhanced gene expression matrix. A variational graph autoencoder model is then used to extract a latent representation. We ran DeepST to obtain latent representations with 200 PCs in this study. Other parameters were kept at their default settings.

***STAGATE***. STAGATE combines an autoencoder with graph attention to learn a latent representation. For the preprocessing step, we used the default settings of 3000 for the top variable genes, normalizing the total expression of each spot to 10000, followed by log transform. Thereafter, the STAGATE model was run with default parameters as suggested in the vignettes.

**Evaluation metrics for clustering**

For datasets with manually annotated spatial domain labels (e.g., DLPFC), 6 metrics were used to measure the performance of different clustering algorithms. The metrics are adjusted rand index (ARI), adjusted mutual information (AMI), purity score, homogeneity score, completeness, and v_measure.

ARI calculates the similarity between the clustering labels predicted by the algorithm and reference cluster labels as:

$$ARI= \frac{RI-E[RI]}{\max\left( RI \right)-E\left[ RI \right]} ,$$

where the ﻿unadjusted rand index (RI) is defined as $RI=(a+b)/C_{n}^{2}$, with $a$ being ﻿the number of pairs correctly labeled as coming from the same set, $b$ being the number of pairs correctly labeled as not in the same set, and $C_{n}^{2}$ being the total number of possible pairs. $E[RI]$ is the expected $RI$ of random labeling. A higher ARI score indicates better performance.

Mutual information (MI) measures the similarity between ground truth and predicted clusters. It is defined as:

$$MI\left( U, V \right)=\sum_{i=1}^{\left| U \right|} \sum_{j=1}^{\left| V \right|} \frac{\left| U_{i}\cap V_{j} \right|}{N}\log\frac{N\left| U_{i}\cap V_{j} \right|}{\left| U_{i} \right|\left| U_{j} \right|}$$

where $\left| U_{i} \right|$ is the number of the samples in cluster $U_{i}$ and $\left| V_{i} \right|$ is the number of the samples in cluster $V_{i}$. MI is generally higher for clustering results with larger number of clusters. To account this bias, the adjusted mutual information (AMI) was calculated to remove the effect of cluster numbers:

$$AMI\left( U, V \right)=\frac{MI\left( U,V \right)-E(MI\left( U,V \right))}{avg\left( H\left( U \right), H\left( V \right) \right)-E(MI(U, V))}$$

To calculate purity score, we first assign a label to each cluster based on the most frequent class in it. Then the purity score is the number of correctly assigned classes and cluster labels divided by the total amount of data.

A clustering result satisfies homogeneity if all clusters contain only data points which are members of a single class, while a clustering result satisfies completeness if all the data points that are members of a given class are elements of the same cluster. Homogeneity score and completeness score are both defined using Shannon’s entropy:

$$h=\frac{MI(U,V)}{H(U)}$$

$$c=\frac{MI(U, V)}{H(V)}$$

Where MI is mutual information and H is Shannon’s entropy.

V-measure is the harmonic mean value for homogeneity and completeness. The function to calculate V-measure is:

$$v=\frac{Homogeneity*Completeness}{Homogeneity+Completeness}$$

All of the metrics generate values ranged from 0 to 1 and a higher score indicates better performance for clustering.

Silhouette score is a metric used to calculate the goodness of a clustering method. It is defined as $\frac{b-a}{max\left( a-b \right)}$, where a is the mean intra-cluster distance and b is the mean nearest-cluster distance. However, the input of clustering is the different latent representation and clustering output of methods, which makes the comparison meaningless. To make the comparison more reasonable, we used the latent representation of each method as feature matrix and used the ground truth as the ‘pseudo cluster label’ to calculate Silhouette score. Silhouette score is then used to measure the goodness of matching between latent representations of different methods and the ground truth.

**Several downstream analyses**

After running SEDR on spatial transcriptomics data, low dimensional embeddings and spot clusters are obtained. In this manuscript, several downstream analyses have bseen done to evaluate SEDR.

***Use Monocle 3 to do trajectory analysis***

On the DLPFC #151673 slice and breast cancer data, Monocle 3 was run with the latent representations provided by the clustering methods. We used the uwot package to calculate UMAP from the latent representations. Setting white matter (WM) as the starting point, Monocle 3 used the UMAPs to generate pseudo-time trajectory with recommended parameters.

***Use Harmony to correct batch effect***

Harmony was used to correct for batch effects in low-dimensional embeddings. Using the latent representations as input, Harmonypy (v0.0.9) was used to integrate it across the 12 DLPFC sections. The integration results were then used for UMAP visualization and quantitative analysis, such as LISI. LISI was calculated by compute_lisi function in Harmonypy (v0.0.9). There are two types of LISI score, one is cLISI that is calculated with cell types, while another is iLISI which is calculated with datasets. Higher cLISI means the neighbors of cell/spot contain more types of clusters, while higher iLISI means the neighbors of cell/spot come from more datasets. Therefore, low cLISI score and high iLISI score indicate better integration. Since the value of cLISI and iLISI depends on the total number of clusters or datasets, we normalize them to [0, 1], and make the value the larger the better by following equation:

$$n(cLISI)=\frac{N_{cell types}-cLISI}{N_{cell types}-1}$$

$$n(iLISI)=\frac{iLISI-1}{N_{datasets}-1}$$

where $N_{cell types}$ is the number of clusters and $N_{datasets}$ is the number of datasets.

**Methods used for breast cancer analysis**

***Prediction of cell type composition of 10x Genomics Visium spatial spots***

We downloaded a published scRNA-seq dataset of human breast cancer [12] as reference and ran Seurat to find transfer anchors between the reference and the Visium spatial data. Cell types in the reference were then assigned to the spatial spots by label transfer. We removed cell types with probabilities equal to 0 for all spots.

***Differential expression analysis*** ***and pathway analyses***

We used Seurat to identify DEGs. Differentially expressed genes with an adjusted p-value < 0.05 were used as the input for QIANGEN Ingenuity Pathway Analysis (IPA). For the IPA results, pathways with positive or negative z-scores were plotted.

**References**

1. Dobin A, Davis CA, Schlesinger F, Drenkow J, Zaleski C, Jha S, Batut P, Chaisson M, Gingeras TR: **STAR: ultrafast universal RNA-seq aligner.** *Bioinformatics* 2013, **29:**15-21.

2. Hao Y, Hao S, Andersen-Nissen E, Mauck WM, 3rd, Zheng S, Butler A, Lee MJ, Wilk AJ, Darby C, Zager M, et al: **Integrated analysis of multimodal single-cell data.** *Cell* 2021, **184:**3573-3587 e3529.

3. Chen Z, Soifer I, Hilton H, Keren L, Jojic V: **Modeling Multiplexed Images with Spatial-LDA Reveals Novel Tissue Microenvironments.** *J Comput Biol* 2020, **27:**1204-1218.

4. Dries R, Zhu Q, Dong R, Eng CL, Li H, Liu K, Fu Y, Zhao T, Sarkar A, Bao F, et al: **Giotto: a toolbox for integrative analysis and visualization of spatial expression data.** *Genome Biol* 2021, **22:**78.

5. Pham D, Tan X, Xu J, Grice LF, Lam PY, Raghubar A, Vukovic J, Ruitenberg MJ, Nguyen Q: **stLearn: integrating spatial location, tissue morphology and gene expression to find cell types, cell-cell interactions and spatial trajectories within undissociated tissues.** *bioRxiv* 2020**:**2020.2005.2031.125658.

6. Liu Q, Hsu CY, Shyr Y: **Scalable and model-free detection of spatial patterns and colocalization.** *Genome Res* 2022, **32:**1736-1745.

7. Hu J, Li X, Coleman K, Schroeder A, Ma N, Irwin DJ, Lee EB, Shinohara RT, Li M: **SpaGCN: Integrating gene expression, spatial location and histology to identify spatial domains and spatially variable genes by graph convolutional network.** *Nat Methods* 2021.

8. Kim J, Rustam S, Mosquera JM, Randell SH, Shaykhiev R, Rendeiro AF, Elemento O: **Unsupervised discovery of tissue architecture in multiplexed imaging.** *Nat Methods* 2022, **19:**1653-1661.

9. Zhao E, Stone MR, Ren X, Guenthoer J, Smythe KS, Pulliam T, Williams SR, Uytingco CR, Taylor SEB, Nghiem P, et al: **Spatial transcriptomics at subspot resolution with BayesSpace.** *Nat Biotechnol* 2021.

10. Xu C, Jin X, Wei S, Wang P, Luo M, Xu Z, Yang W, Cai Y, Xiao L, Lin X, et al: **DeepST: identifying spatial domains in spatial transcriptomics by deep learning.** *Nucleic Acids Res* 2022, **50:**e131.

11. Dong K, Zhang S: **Deciphering spatial domains from spatially resolved transcriptomics with an adaptive graph attention auto-encoder.** *Nat Commun* 2022, **13:**1739.

12. Pal B, Chen Y, Vaillant F, Capaldo BD, Joyce R, Song X, Bryant VL, Penington JS, Di Stefano L, Tubau Ribera N, et al: **A single-cell RNA expression atlas of normal, preneoplastic and tumorigenic states in the human breast.** *EMBO J* 2021, **40:**e107333.

**Supplementary figures**

**
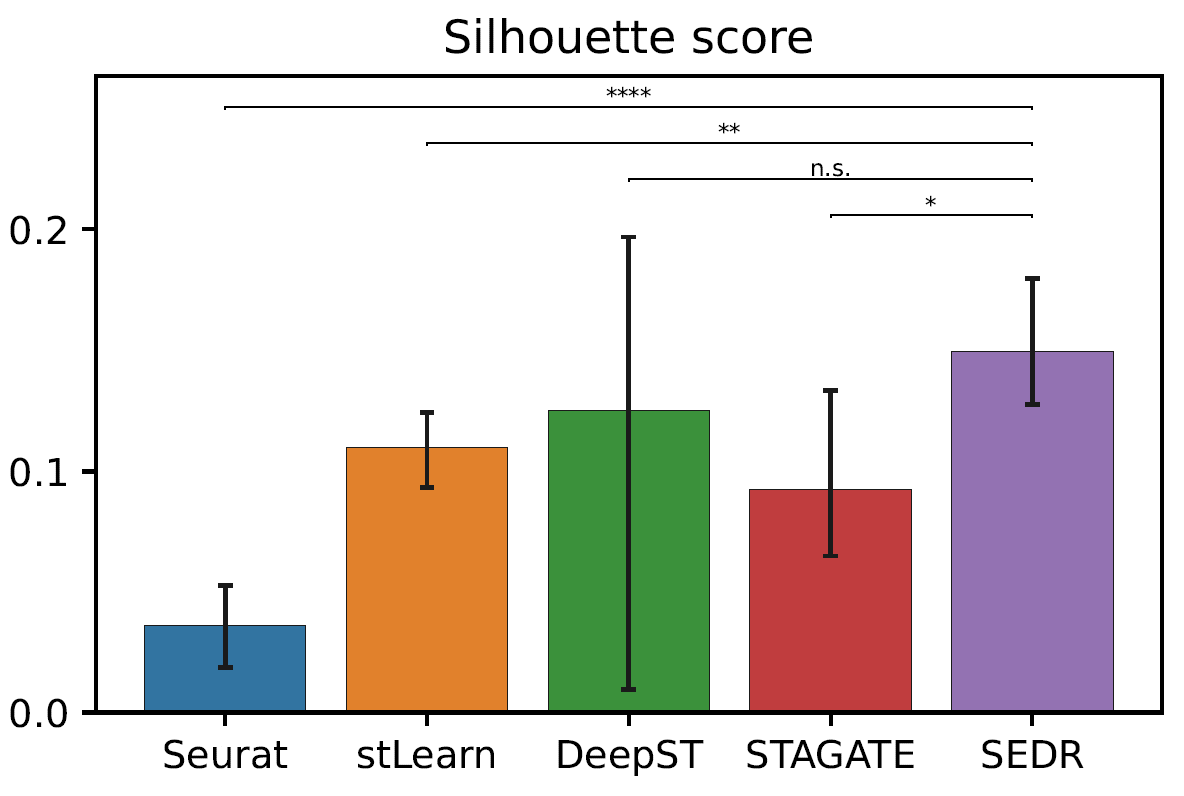
Fig S1. Silhouette score for 5 methods on 12 DLPFC sections.** n.s.: p-value>0.05, *: p-value<0.05, **:p-value<0.005, ***:p-value<0.0005, ****:p-value<0.00005


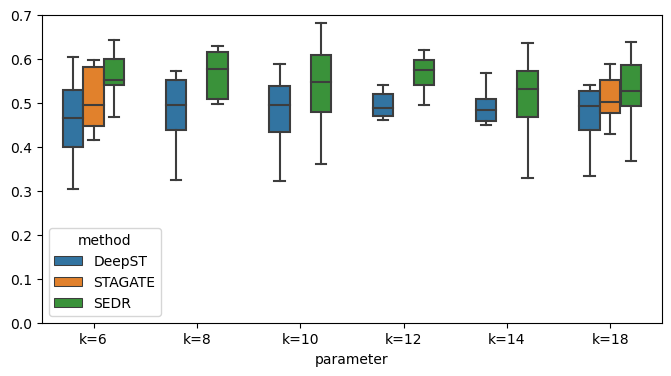


Fig S2. ARI boxplot for 3 methods on 12 DLPFC datasets with different K (number of nearest neighbor).


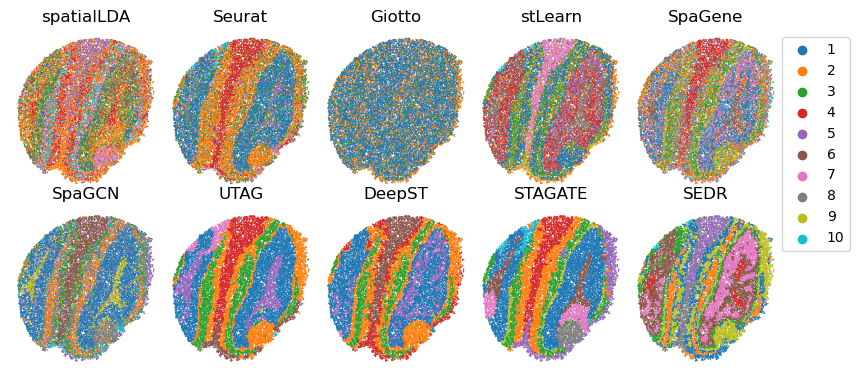


Fig S3. Unsupervised clustering results for SEDR and competing methods on olfactory bulb Slide-seq data.

**
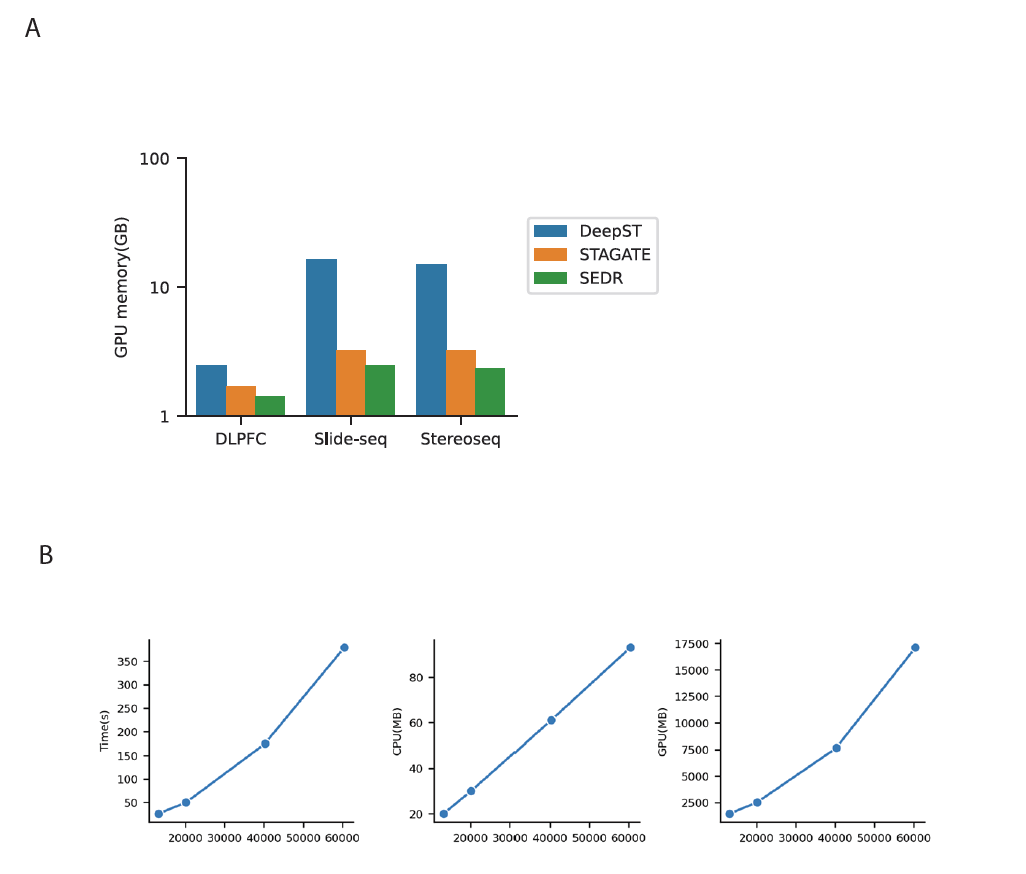
**

Fig S4. Computational requirements of SEDR.

1. GPU memory usage for DeepST, STAGATE and SEDR when processing DLPFC, mouse olfactory bulb Slide-seq and Stereo-seq data.
2. Time, CPU memory and GPU memory costed by SEDR on simulated data. Simulated data is generated with Slide-seq data (20000 spots) by randomly selecting ½, 1, 2, 4 times of the spots as the raw data.


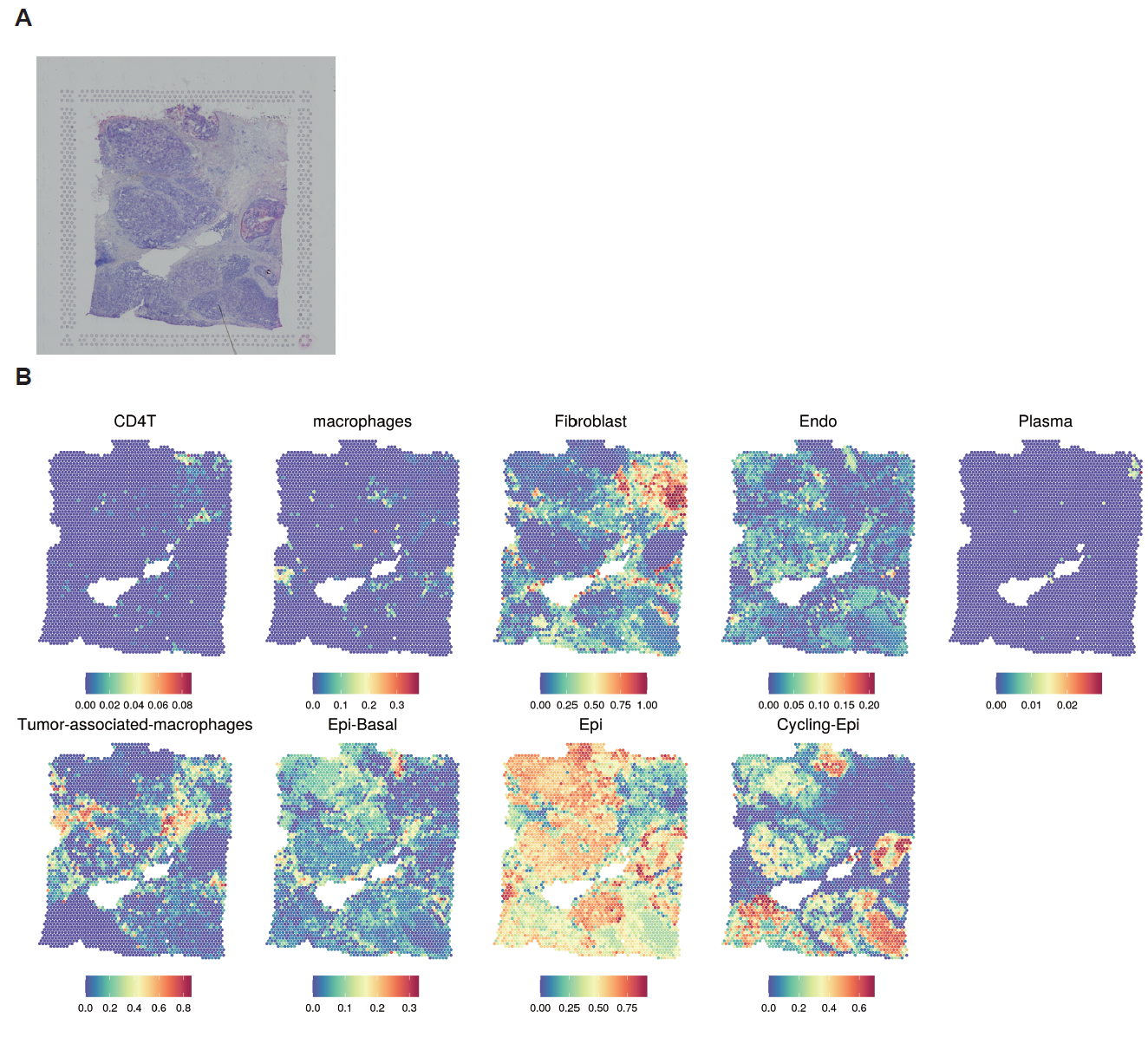


Fig S5. Human breast cancer histology and cell type mixtures of spatial spots.

1. H&E staining.
2. Probability of cell types for spots that was predicted by Seurat.


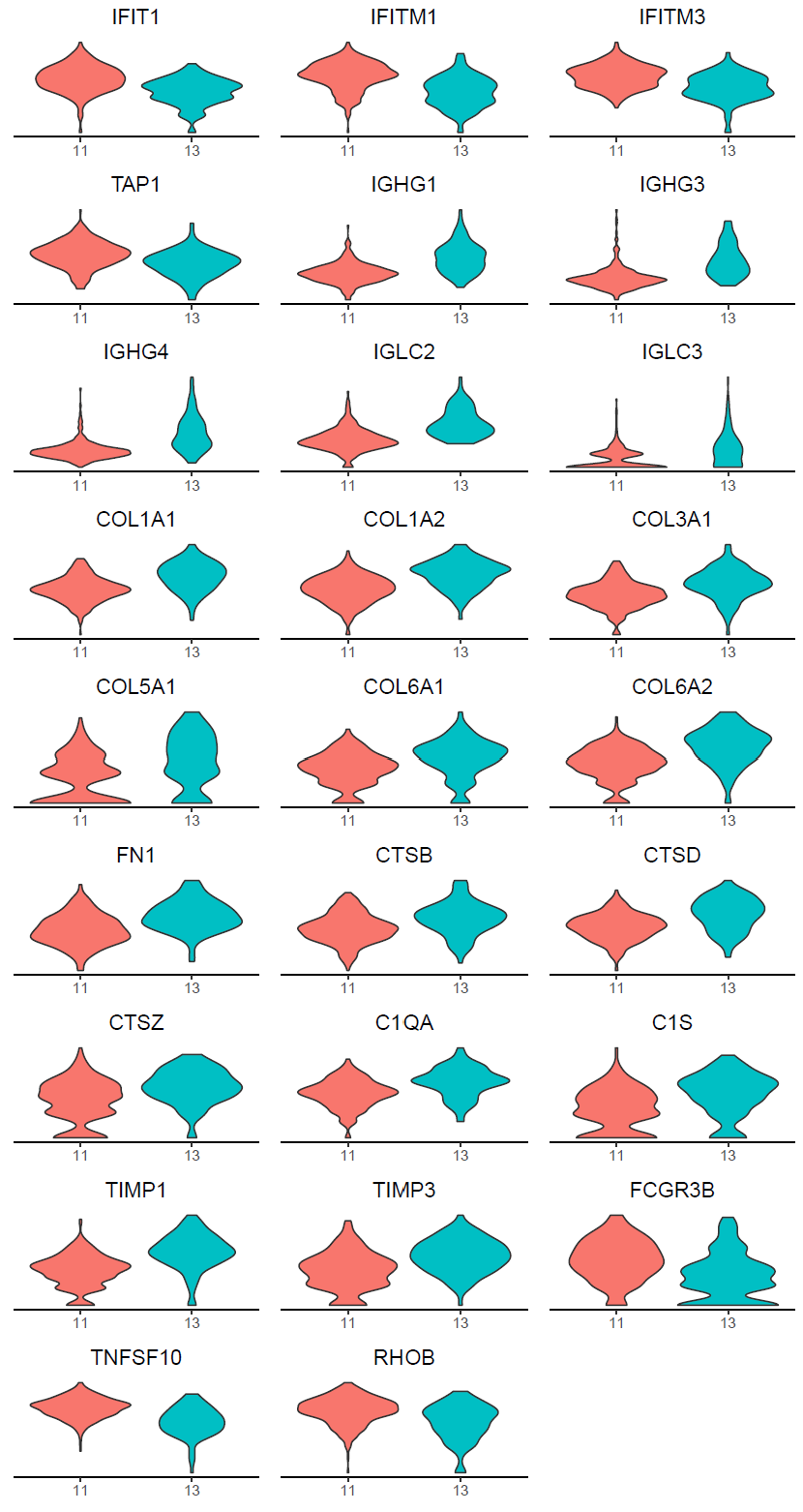


Fig S6. Differentially expressed genes (DEGs) between SEDR cluster 11 and cluster 13 in human breast cancer data.
